# Psychiatric comorbidity in a outpatient sample with Parkinson’s disease

**DOI:** 10.1192/j.eurpsy.2025.734

**Published:** 2025-08-26

**Authors:** G. Öksüz Bildirici, E. Sönmez Güngör

**Affiliations:** 1 Psychiatry, Erenkoy Mental Health and Neurological Diseases Training and Research Hospital, Istanbul, Türkiye

## Abstract

**Introduction:**

Parkinson’s disease (PD) is a progressive neurodegenerative disorder with a wide variety of clinical symptoms such as tremor, stiffness, and slowness. Additionally, prodromal features, psychological or cognitive problems may be observed which may complicate the treatment.

**Objectives:**

In this study, we aimed to investigate the rate and extent psychiatric comorbidity (PC) in PD patients admitting to psychiatric outpatient clinic.

**Methods:**

A retrospective, cross-sectional study was designed. Electronic medical records were examined for data collection of all outpatients admitting to Erenköy Mental Health and Neurological Diseases Training and Research Hospital outpatient psychiatric clinics between June 1, 2023, and June 1, 2024 who had an ICD diagnosis of G20, G21, and G22, along with their subcategories. Informed consent was obtained.The findings were grouped according to PC.

**Results:**

Out of 37 533 outpatients, 61 outpatients (27 male, 34 female) of them were matching the inclusion criteria. Three of them had no PC and the prevalence of PC in PH was found as 95%. The patients presented with PC were chronologically categorized into 3 groups. Each of these categories was further divided into 6 subcategories based on ICD codes (F10–19, F20–29, F30–39, F40–48, G47.0–47.9, others) (See Table 1). Of note, 3 of 61 patients (4.9%) had dopamine dysregulation syndrome (DDS), characterized by addictive use of dopaminergic drugs, mood swings, compulsive and impulsive behaviors and psychosis (1).

Despite we have chronologically grouped the sample, it is not possible to determine with certainty whether the PC diagnosed before PD is a prodromal symptom or a seperate diagnosis.

Since the study was conducted at a mental health hospital, the prevalence of PC in our sample was higher than what is reported in the literature, which was expected due to sampling biases.

**Image 1:**

**Figure:**
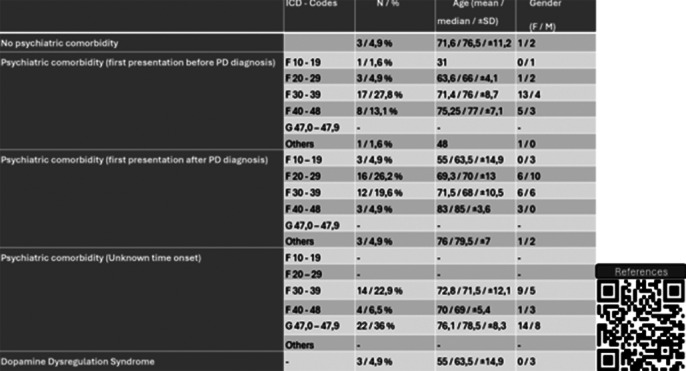

**Conclusions:**

PC is common among PD patients. It may present as prodromal symptoms of PD, may occur due to disease or medication after the onset of PD, or primary neurological and psychiatric conditions may be concomitant.

PC are often overlooked in the diagnosis and treatment and this situation complicates treatment and increases burden. Multidisciplinary approach is essential.

In our study, the highest prevalence of PC being F 30–39 is consistent with the literature. It has been determined that the most common psychiatric disorders in PD are depression (31%) (2,3).

It is noted that the most common PC before and after the diagnosis of PD were F30–39 and F20–29, respectively. It is compatible with literature. PD may present prodromal symptoms such as depression, on the contrary, it may progress with psychotic symptoms. The prevalence of psychosis in PD varies between 43-60% (4).

Special attention should be given to DDS, which is 4,9% and consistent with the literature in our sample (The estimated prevalence of 3.4–4%) (5,6). DDS is a very challenging condition to treat.

**Disclosure of Interest:**

None Declared

